# Draft Genome Sequence of *Curtobacterium flaccumfaciens* Strain UCD-AKU (Phylum *Actinobacteria*)

**DOI:** 10.1128/genomeA.00244-13

**Published:** 2013-05-16

**Authors:** Jennifer C. Flanagan, Jenna M. Lang, Aaron E. Darling, Jonathan A. Eisen, David A. Coil

**Affiliations:** University of California Davis, Genome Center, Davis, California, USA

## Abstract

Here we present the draft genome of an actinobacterium, *Curtobacterium flaccumfaciens* strain UCD-AKU, isolated from a residential carpet. The genome assembly contains 3,692,614 bp in 130 contigs. This is the first member of the *Curtobacterium* genus to be sequenced.

## GENOME ANNOUNCEMENT

Members of the *Curtobacterium* genus are obligate aerobes and have been previously isolated from cheese vats (1), soil (2), and numerous plants. *Curtobacterium* is characterized as Gram-positive rods and usually appears in yellow- or orange-colored colonies (3). *Curtobacterium flaccumfaciens* is a recognized and widespread plant pathogen, particularly in members of the bean family (4). Some strains have been isolated from humans (5), and at least one strain of *C. flaccumfaciens* has been shown to be infectious in humans (6).

*Curtobacterium flaccumfaciens* strain UCD-AKU was isolated from a residential carpet in Davis, California, as part of a project to produce reference genomes for microorganisms living in the built environment (7, 8). Carpet fibers were placed in Luria broth (LB), incubated overnight at 37°C, and plated on LB agar. Colonies were isolated by serial dilution streaking. This isolate was identified by sequencing the 16S rRNA gene PCR product produced by the 1391R and 27F primers. Genomic DNA was extracted using a Wizard genomic DNA purification kit (Promega) from a fresh overnight culture.

Two Illumina paired-end libraries were generated using a TruSeq DNA Sample Prep v2 kit (Illumina) and a Nextera DNA Sample Prep kit (Illumina). We selected 300- to 600-bp fragments using a Pippin Prep (Sage Science). Libraries were sequenced on an Illumina MiSeq, with a read length of 250 bp, trimmed to 160 bp prior to assembly. This produced a total of 6,705,982 paired-end reads. Quality trimming and error correction of the reads resulted in 6,042,026 high-quality reads. These steps were performed using the a5 assembly pipeline (9). This pipeline automates data cleaning, error correction, contig assembly, scaffolding, and quality control. An additional assembly was generated using the CLC Workbench (CLC Bio). The two assemblies were mapped to each other using progressiveMauve (10) and scaffolds from the CLC assembly not present in the A5 assembly were removed. The resulting “consensus” assembly contained 38 scaffolds (minimum, 446 bp; maximum, 630,288 bp; N_50_, 233,227). During scaffolding, some contigs were merged based on short overlaps and read pair information, yielding a final collection of 130 contigs that were submitted to GenBank. This final assembly had 3,692,614 bp with a GC content of 71% and a coverage estimate of 261×. Genome completeness was assessed using the PhyloSift software (A. Darling, G. Jospin, E. Lowe, E. Matsen, H. Bik, and J. Eisen, submitted), which searches for a list of 40 highly conserved, single-copy marker genes (D. Wu, G. Jospin, and J. Eisen, unpublished data), of which all were found in this assembly.

Annotation was performed using the RAST server (11). *C. flaccumfaciens* UCD-AKU contains 3,462 predicted protein-coding sequences and 50 predicted noncoding RNAs. Identification of arsenic resistance genes is consistent with previous experimental studies (12).

A phylogenetic tree of 16S rRNA gene sequences from cultured isolates of *Curtobacterium* was produced using the Ribosomal Database Project (RDP), which implements a weighted neighbor-joining algorithm (13). *C. flaccumfaciens* UCD-AKU falls within a well-supported (99% bootstrap support) clade that contains only *C. flaccumfaciens* isolates (doi: 10.6084/m9.figshare.646183).

### Nucleotide sequence accession numbers.

This whole-genome shotgun project has been deposited at DDBJ/EMBL/GenBank under the accession number APJN00000000. The version described in this paper is the first version, APJN01000000. Illumina reads are available at doi:10.6084/m9.figshare.644657.
